# Gelseminum as an Antiperiodic

**Published:** 1873-11

**Authors:** William W. Murray

**Affiliations:** Professor of Materia Medica and Therapeutics, in the College of Physicians and Surgeons, Baltimore


### GELSEMINUM AS AN ANTIPERIODIC.

BY WILLIAM W. MURRAY, M. D.

Professor of Materia Medica and Therapeutics, in the College of Physicians and Surgeons, Baltimore.

Since the accidental discovery of the febrifuge virtues of Gelseminum, its use in febrile and inflammatory diseases has been known to the profession. From some it has received the highest encomiums as an anti-phlogistic and febrifuge; by others it is but little esteemed. While I am among those who think well of this drug in its power of reducing the inordinate action of the heart in inflammatory affections, it is not the object of this paper to discuss its virtues in diseases of that kind, but I desire to call the attention of the profession to its *antiperiodic* powers.

I have not had access to all the articles which have been written on the medicinal properties and uses of Gelseminum, but so far as I am aware, its antiperiodic effects were first made public by Dr. E. A. Anderson, of Wilmington, N. C., in an article which appeared in "Tilden's Journal of Materia Medica," of 1780, either the October or November number. I need not refer to Dr. Anderson's paper, or to a private communication from him on the subject, further than to say that by the strength of his assertions that Gelseminum will radically cure malarial fevers, I was induced to make use of it in those affections, in order to satisfy myself whether it was entitled to take rank as an *antiperiodic.* While the cases in which I have employed it are not very numerous, the results were of such a character as to convince *me,* at least, that it is not only equal to quina in breaking in upon the chain of morbid phenomena which characterize intermittent fever, but that it is infinitely superior to that article in curing the disease. It is tasteless and cheap, both of which are additional advantages which it possesses over quinia.

On May 14th, 1872, I was called to see Mrs. B., who was laboring under an attack of intermittent fever, tertian form. I determined to put her on Gelseminum, especially as she was pregnant, in which condition quinia is considered, by some physicians, somewhat dangerous. Accordingly after opening the bowels and appealing to the liver, I prescribed the tincture of yellow jasmine in five-minim doses every hour, until it produced heaviness of the eye-lids, dilated pupils or double vision ; the same course to be pursued for four or five days successively. She has had no return of the disease in any form since, although she passed through the entire miasmatic season in the same locality.

The next case of intermittent fever which fell to my care was that of Mrs. P., on the 24th of May. She had suffered frequently from this affection, and she implored me not to give her quinia, as it affected her very distressingly. She seemed as much surprised as delighted when I informed her that I had no intention of giving the much dreaded medicine. I ordered tinct. Gelsemini in the same doses, and with the same directions as in the preceding case. She has had no chill since then to the present, Jan. 3d, 1873.

On 5th July, 1873, I was called to see a little boy, whom I found laboring under an attack of remittent fever. Gelseminum was ordered for him, and on the third day my visits ceased, the disease having entirely yielded.

On the 14th July I visited Miss P., who had remittent fever. As the case presented some pretty severe symptoms, and not wishing to risk anything (knowing that the quinia treatment, if properly managed was endorsed by experience), I urged quinia, which she consented to take after a great deal of persuasion, (she had taken it before in a similar attack, and as she said, "it nearly set her crazy,") but she had swallowed only a few grains, when it affected her so unpleasantly that she could not be induced to take any more. I then ordered gelseminum, which cured her. The disease, in this case, did not yield so readily as in that of the little boy referred to, for two reasons: 1st, it was a much more severe attack; and 2d, she would not continue the medicine faithfully, according to directions, but as soon as she began to feel better under its use, she would cease taking it. As regarded the antiperiodic treatment, it was the only drug used, and she made an excellent recovery.

The last case which I will mention is that of Miss C., who had tertain ague. Quinia had always arrested the paroxysms, but only temporarily. I gave her blue pill and compound ext. colocynth, each gr. vj., and then put her on the yellow jasmine. This was about four months ago, since which time she has had no return of the affection.

I may remark that in the use of the gelseminum, I am governed by the effect produced, whether the quantity required to produce that effect be small or great. I give it generally in five-minim doses, and I think this the safest plan, because the effects can be regulated exactly; whereas, if large doses be given, we may have an overwhelming effect produced at once, which would be at least alarming, and possibly dangerous.

I have been induced to narrate these cases, in the hope that some of the readers of the Reporter will test for themselves the antiperiodic virtues of gelseminum, and record the results of their experience for the benefit of the profession at large.—*Philadelphia Med. and Surg. Reporter.*

